# Incident comorbidities in patients with chronic hypoparathyroidism after thyroidectomy: a multicenter nationwide study

**DOI:** 10.3389/fendo.2024.1348971

**Published:** 2024-02-28

**Authors:** Juan J. Díez, Emma Anda, Begoña Pérez-Corral, Miguel Paja, Victoria Alcázar, Cecilia Sánchez-Ragnarsson, Aida Orois, Ana R. Romero-Lluch, Marcel Sambo, Amelia Oleaga, Águeda Caballero, María R. Alhambra, Virginia Urquijo, Ana M. Delgado-Lucio, José C. Fernández-García, Viyey Kishore-Doulatram, Suset Dueñas-Disotuar, Tomás Martín, Mercedes Peinado, Julia Sastre

**Affiliations:** ^1^ Department of Endocrinology, Hospital Universitario Puerta de Hierro Majadahonda, Instituto de Investigación Sanitaria Puerta de Hierro Segovia de Arana, Majadahonda, Spain; ^2^ Department of Medicine, Universidad Autónoma de Madrid, Majadahonda, Spain; ^3^ Department of Endocrinology, Hospital Universitario de Navarra, Pamplona, Spain; ^4^ Department of Endocrinology, Complejo Asistencial Universitario de León, León, Spain; ^5^ Department of Endocrinology, Hospital Universitario de Basurto, Universidad del País Vasco (UPV/EHU), Bilbao, Spain; ^6^ Department of Endocrinology, Hospital Severo Ochoa, Leganés, Spain; ^7^ Department of Endocrinology, Hospital Universitario Central de Asturias, Instituto de Investigación Sanitaria del Principado de Asturias, Oviedo, Spain; ^8^ Department of Endocrinology and Nutrition, Hospital Clínic, Barcelona, Spain; ^9^ Department of Endocrinology, Hospital Universitario Virgen del Rocío, Sevilla, Spain; ^10^ Department of Endocrinology, Hospital General Universitario Gregorio Marañón, Madrid, Spain; ^11^ Department of Endocrinology, Hospital Universitario de Canarias, Tenerife, Spain; ^12^ Department of Endocrinology, Hospital Universitario Reina Sofía, Córdoba, Spain; ^13^ Department of Endocrinology, Hospital Universitario de Cruces, Bilbao, Spain; ^14^ Department of Endocrinology, Hospital Universitario de Burgos, Burgos, Spain; ^15^ Department of Endocrinology, Hospital Regional Universitario de Málaga, Instituto de Investigación Biomédica de Málaga, Universidad de Málaga, Málaga, Spain; ^16^ Department of Endocrinology, Hospital Universitario Virgen Macarena, Sevilla, Spain; ^17^ Department of Endocrinology, Hospital Universitario de Toledo, Toledo, Spain

**Keywords:** postsurgical hypoparathyroidism, comorbidity, incidence, prevalence, thyroidectomy

## Abstract

**Purpose:**

Population-based and registry studies have shown that chronic hypoparathyroidism is accompanied by long-term complications. We aimed to evaluate the risk of incident comorbidity among patients with chronic postsurgical hypoparathyroidism in real-life clinical practice in Spain.

**Methods:**

We performed a multicenter, retrospective cohort study including patients with chronic postsurgical hypoparathyroidism lasting ≥3 years with at least a follow-up visit between January 1, 2022 and September 15, 2023 (group H). The prevalence and incidence of chronic complications including chronic kidney disease, nephrolithiasis/nephrocalcinosis, hypertension, dyslipidemia, diabetes, cardiovascular disease, central nervous system disease, mental health disorders, eye disorders, bone mineral density alterations, fracture and cancer were evaluated. Patient data were compared with a group of patients who did not develop hypoparathyroidism, matched by gender, age, and follow-up time after thyroidectomy (group NH).

**Results:**

We included 337 patients in group H (median [IQR] age, 45 [36-56] years; median time of follow-up, 8.9 [6.0-13.0] years; women, 84.3%) and 669 in group NH (median age, 47 [37-55] years; median time of follow-up, 8.0 [5.3-12.0] years; women, 84.9%). No significant differences were found in the prevalence of comorbidities at the time of thyroidectomy between both groups. In multivariable adjusted analysis, patients with chronic hypoparathyroidism had significantly higher risk of incident chronic kidney disease (OR, 3.45; 95% CI, 1.72-6.91; P<0.001), nephrolithiasis (OR, 3.34; 95% CI, 1.55-7.22; P=0.002), and cardiovascular disease (OR, 2.03; 95% CI, 1.14-3.60; P=0.016), compared with patients without hypoparathyroidism. On the contrary, the risk of fracture was decreased in patients with hypoparathyroidism (OR, 0.09; 95% CI, 0.01-0.70; P=0.021).

**Conclusion:**

This study demonstrates that, in the clinical practice of Spanish endocrinologists, a significant increase in the risk of chronic kidney disease, nephrolithiasis and cardiovascular disease, as well as a reduction in the risk of fractures is detected. These results are of interest for the development of new clinical guidelines and monitoring protocols for patients with hypoparathyroidism.

## Introduction

Hypoparathyrodism is a rare endocrine disorder characterized by absence or inappropriately low levels of parathyroid hormone leading to hypocalcemia and hyperphosphatemia (1). In approximately 75% of cases it occurs as a complication of anterior neck surgery and is therefore seen more frequently in older adult women (2). Postsurgical hypoparathyroidism is a result of inadvertent removal or injury of the parathyroid glands during thyroid or parathyroid surgery. Hypoparathyroidism may be transient, lasting several days or weeks, protracted, lasting some months, or permanent (1, 3, 4). Hypoparathyroidism is considered definitive or permanent when the need for treatment with calcium and active vitamin D metabolites lasts more than 6 months (3, 4) or 12 months after surgery (5–7). The prevalence of permanent hypoparathyroidism varies depending on patient characteristics, diagnostic criteria, surgical experience and geographical area (8). Based on data from reviews and meta-analyses, the prevalence of postoperative persistent hypoparathyroidism had been estimated to be 0-3% (8). However, more recent studies conducted using national registries (9, 10) or multicenter cohort analyses (6, 11, 12) have shown prevalence values between 11-28%. In the particular case of Spain, a multicenter, nationwide study, carried out in the setting of clinical practice, in reference centers, showed that the prevalence of definitive hypoparathyroidism among patients with total thyroidectomy was 14.5% (6). All these data emphasize the potential importance of this hormonal deficiency in patients undergoing thyroid surgery.

Main goals of management of patient with hypoparathyroidism include normalization of calcium and phosphate metabolism parameters and preventing signs and symptoms of hypocalcemia and hypercalcemia (1). An additional objective in long-term follow-up should be the prevention of complications and comorbidities (3, 4). It has been reported that patients with hypoparathyroidism have a high risk of developing kidney failure, kidney stones, neuropsychiatric disease, epilepsy and cataracts in comparison with normal population (13–15). More recent studies have found that hypoparathyroidism is also associated with cardiovascular disease, infections and even an increased risk of mortality (16). The association of hypoparathyroidism with fractures is controversial, since variable results have been found in different studies (16, 17). Furthermore, a study showed that the risk of gastrointestinal cancer was significantly reduced in patients with postsurgical hypoparathyroidism (14).

Most authors who have associated chronic hypoparathyroidism with various comorbidities have carried out population-based studies or have used data from large national registries. To our knowledge, no clinical series of patients with hypoparathyroidism or studies that use data from routine clinical practice have been published to elucidate the risks of comorbidity in these patients in real life. Hence, in this study we aimed to compare the appearance of different comorbidities diagnosed after total thyroidectomy in a group of patients with long-term definitive hypoparathyroidism with those found in a similar group of thyroidectomized patients without hypoparathyroidism.

## Methods

### Subjects

This is a multicenter, retrospective cohort study, with data from routine clinical practice, performed in patients treated by total thyroidectomy for any cause with a follow-up time of at least three years after surgery. We included all patients with permanent hypoparathyroidism lasting at least 3 years (group H) who attended the endocrinology clinics of the participating hospitals during the study period. For comparison purposes, we also analyzed a control group of patients who did not develop permanent hypoparathyroidism after surgery (group NH). For each patient, 1-3 controls matched by sex, age, and follow-up time after thyroidectomy were selected.

Inclusion criteria for patients with and without hypoparathyroidism were the following: age ≥18 years at the time of total thyroidectomy (one or two stages), availability of histological report, follow-up in the same hospital for a period ≥3 years, and date of last visit between January 1, 2022 and September 15, 2023.

### Study design

This project was disseminated through the Thyroid Task Force of the Spanish Society of Endocrinology and Nutrition (Sociedad Española de Endocrinología y Nutrición, SEEN) composed of endocrinologists with special expertise and dedication to thyroid disease. Twenty investigators from 16 hospital centers agreed to participate in the study. A review of the medical records of all patients who met the inclusion criteria was performed. Each investigator selected patients with hypoparathyroidism who met the inclusion criteria and with at least one follow-up visit during the recruitment period.

We collected information on clinical and demographic data, initial surgery, pathological details, prevalent chronic diseases before thyroidectomy, follow-up time after surgery, and incident diseases detected in clinical practice until the last visit. For the study of prevalence and incidence, the following conditions were considered: chronic kidney disease (stage 3 or higher, i.e., estimated glomerular filtration rate <60ml/min/1.73m^2^), nephrolithiasis/nephrocalcinosis, hypertension, dyslipidemia, diabetes, cardiovascular disease, central nervous system disease, mental health disorders, eye disorders, bone mineral density (BMD) alterations, fracture and cancer. The usual criteria were used for the diagnosis of these procedures in clinical practice and the presence of the diagnoses was verified in the patients’ medical record. The glomerular filtration rate was estimated by the usual method in each of the participating hospitals (CDK-EPI equation (18) in 58% of the subjects and MDRD 4-variable equation (19) in 42%). For each patient we recorded all prevalent diseases at the time of thyroidectomy and all incident diseases, with the date of diagnosis, from thyroidectomy to the end of follow-up. We also registered the chronic pharmacological treatments used by patients both at the time of thyroidectomy and at the last follow-up visit.

All patient’s data were obtained under the standard medical care conditions. The patient’s confidential information was protected according to national law, and the study received favorable report from the ethics committee of the Hospital Universitario Puerta de Hierro Majadahonda (PI 253/22).

### Statistical analysis

For quantitative variables, results are expressed as median (interquartile range, IQR). Categorical variables are described as absolute values, ratios, or percentages. For proportion comparisons, the chi-square test or Fisher’s exact test was used. The McNemar test was used to compare the proportions of drug use at the time of thyroidectomy and at the last follow-up visit (paired data). For the analysis of incident comorbidities we estimated the values (with the 95% confidence interval) for the cumulative incidence and incidence rate in patients in group H. For the NH group we estimated the proportion of patients who developed incident diseases. For this analysis, the risk of each comorbidity was assessed among patients free of that condition during the baseline period.

Cumulative incidence (%) was calculated as the number of new cases of disease during follow-up divided by the total number of individuals in the population at risk at the beginning of follow-up. Incidence rate (cases per 100 patient-years) was estimated as the number of new cases of disease divided by the sum of the individual observation times of the at-risk population. To analyze the risk of developing comorbidities in patients with hypoparathyroidism we estimated the odds ratio (OR) as the ratio between the odds in group H and those obtained in the group NH, along with the corresponding 95% confidence interval.

To assess the association of hypoparathyroidism with the appearance of incident comorbidities, we selected the incident diseases in which a significant increase or decrease in odds ratio was detected in patients with hypoparathyroidism compared to group NH. In these cases, we performed a survival analysis using the Kaplan-Meier method, with the log-rank test used to compare groups. Multivariable logistic regression analysis was conducted to evaluate the relative importance of hypoparathyroidism as well as demographic and clinical characteristics of patients for the development of the different comorbidities. For the multivariable analysis, two models were used. Model 1 was adjusted for hypoparathyroidism, gender, age, thyroidectomy, histopathology, hypertension, dyslipidemia, diabetes, cardiovascular disease, and BMD alterations; model 2 was adjusted for the same covariates, and nephrolithiasis, central nervous system disease, mental health disorders, eye disorders, fracture, and cancer. All used tests were two-sided and differences were considered significant when P < 0.05. SPSS software version 21 was used to perform the statistical analysis.

## Results

### Studied patients

Of the 366 patients with hypoparathyroidism initially selected for the study, 59 were excluded due to lack of clinical data during follow-up. Finally, in group H, 337 patients were included (284 women, 84.3%), aged between 18-80 years (median, 45[36-56] years). One hundred and twelve patients (33.2%) underwent thyroidectomy due to benign thyroid disease (follicular nodular disease in 65.2% of these cases, Graves’ disease in 16.1%, chronic thyroiditis in 12.5% and others in 6.3%) and 225 (66.8%) due to thyroid cancer (papillary in 86.2% of these cases, follicular in 8.0%, oncocytic in 1.3% and others in 4.4%). The median follow-up time after thyroidectomy was 8.9 (6.0-13.0) years. The most common prevalent diseases at the time of thyroidectomy were dyslipidemia (19.3%), hypertension (16.3%), mental health disorders (13.1%), diabetes (4.5%), cancer (5.3%), eye disorders (3.3%), and cardiovascular disease (3.0%). The rest of the comorbidities were detected in less than 3% of the patients (**Table 1**).

**Table 1:** Demographic and clinical characteristics of patients with and without hypoparathyroidism.

	Patients with hypoparathyroidism (Group H, n=337)	Patients without hypoparathyroidism (Group NH, n=669)
**Gender**		
Female	284 (84.3)	568 (84.9)
Male	53 (15.7)	101 (15.1)
**Age (yr)**	45 (36-56)	47 (37-55)
**Time of follow-up (yr)**	8.9 (6.0-13.0)	8.0 (5.3-12.0)
**Total thyroidectomy**		
One-stage	302 (89.6)	567 (84.8)
Two-stage	35 (10.4)	102 (15.2)
**Thyroid histopathology**		
Benign thyroid disease	112 (33.2)	120 (17.9)
Thyroid cancer	225 (66.8)	549 (82.1)
**Benign thyroid disease**		
Graves’ disease	18 (16.1)	23 (19.2)
Follicular nodular disease	73 (65.2)	75 (62.5)
Chronic lymphocytic thyroiditis	14 (12.5)	13 (10.8)
Other	7 (6.3)	9 (7.5)
**Thyroid cancer**		
Papillary	194 (86.2)	476 (86.7)
Follicular	18 (8.0)	45 (8.2)
Oncocytic	3 (1.3)	9 (1.6)
Other	10 (4.4)	19 (3.5)
**Chronic kidney disease**	7 (2.1)	15 (2.2)
**Nephrolithiasis**	4 (1.2)	13 (1.9)
**Arterial hypertension**	55 (16.3)	121 (18.1)
**Dyslipidemia**	65 (19.3)	139 (20.8)
**Diabetes**	15 (4.5)	41 (6.1)
**Cardiovascular disease**	10 (3.0)	25 (3.7)
Coronary heart disease	3 (0.9)	8 (1.2)
Cerebrovascular disease	1 (0.3)	7 (1.0)
Other	7 (2.1)	12 (1.8)
**Central nervous system disease**	6 (1.8)	12 (1.8)
**Mental health disorders**	44 (13.1)	93 (13.9)
**Eye disorders**	11 (3.3)	14 (2.1)
**Bone mineral density alterations**	8 (2.4)	22 (3.3)
**Fracture**	3 (0.9)	6 (0.9)
**Cancer**	18 (5.3)	33 (4.9)

Data are the median (IQR) for quantitative variables. For categorical variables, the absolute value is represented and, in parentheses, the prevalence in patients with hypoparathyroidism and the proportion in patients without hypoparathyroidism.

The percentages of the different types of benign thyroid disease and thyroid cancer are calculated for the total of these histological types in patients with (112 benign and 125 malignant) and patients without hypoparathyroidism (120 benign and 549 malignant).

The majority of patients in group H followed replacement treatment with oral calcium and calcitriol (n=191, 86.4%). There were 41 patients (12.2%) who only required calcitriol and 5 (1.5%) that were treated with only oral calcium. In addition, 138 patients (40.9%) were receiving treatment with vitamin D supplements (**Supplementary Material, Supplementary Table 1**). Control of hypoparathyroidism was generally adequate (**Supplementary Material, Supplementary Table 2**). 88.1% of the patients had serum calcium values equal to or greater than 8.0 mg/dl, 65.5% of them had serum phosphorus values equal to or less than 4.5 mg/dl and 98.5% had a serum calcium-phosphorus product less than 55 mg^2^/dl^2^. 24-hour urinary calcium excretion (quantified in 152 patients) was considered normal (<250 mg/24 h in women, <300 mg/24 h in men) in 73.7% of patients with this parameter available.

The group NH consisted of 669 patients (568 women, 84.9%) aged between 18 and 79 years (median 47[37-55] years). Demographic and clinical characteristics are shown in **Table 1**. We did not observe any differences between groups regarding gender, age, time of follow-up, and type of thyroidectomy. However, the percentage of patients with thyroid cancer was higher in group NH (82.1%) in relation to group H (66.8%; P<0.001). Differences between both groups in the prevalence of the different studied comorbidities were not observed.

### Incident comorbidities during follow-up

The values of cumulative incidence and incidence rate in patients with hypoparathyroidism are summarized in **Table 2** and row data are shown in **Supplementary Table 3 (Supplementary Material)**. Incident comorbidities more commonly diagnosed during follow-up in patients in group H were dyslipidemia (incidence rate 2.14[1.57-2.70] cases per 100 patient-years), mental health disorders (1.61[1.14-2.08] cases per 100 patient-years) and arterial hypertension (1.58[1.11-2.05] cases per 100 patient-years).

**Table 2:** Obtained values of cumulative incidence and incidence rates of incident comorbidities in patients with hypoparathyroidism and proportion of patients without hyperparathyroidism who develop incident comorbidities.

	Patients with hypoparathyroidism (group H)		Patients without hypoparathyroidism (group NH)	Odds ratio	
	Cumulative incidence (%)	Incidence rate (per 100 patient-year)	Proportion (%)	Value	P
**Chronic kidney disease**	7.27 (4.47-10.07)	0.73 (0.44-1.02)	2.60 (1.38-3.82)	2.94 (1.56-5.55)	0.001
**Nephrolithiasis**	5.41 (2.98-7.83)	0.54 (0.29-0.79	1.83 (0.80-2.85)	3.07 (1.46-6.45)	0.003
**Hypertension**	15.25 (11.05-19.44)	1.58 (1.11-2.05)	19.53 (16.21-22.84)	0.74 (0.50-1.09)	0.130
**Dyslipidemia**	20.22 (15.45-24.29)	2.14 (1.57-2.70)	22.08 (18.54-25.61)	0.90 (0.62-1.28)	0.545
**Diabetes**	7.14 (/4.33-9.96)	0.72 (0.43-1.02)	6.69 (4.73-8.64)	1.07 (0.63-1.82)	0.793
**Cardiovascular disease**	8.26 (5.27-11.24)	0.84 (0.52-1.16)	4.35 (2.27-5.92)	1.98 (1.15-3.42)	0.014
**Coronary heart disease**	2.10 (0.56-3.63)	0.21 (0.05-0.36)	1.21 (0.38-2.04)	1.75 (0.63-4.86)	0.285
**Cerebrovascular disease**	2.98 (1.16-4.79)	0.30 (0.11-0.48)	1.36 (0.48-2.24)	2.23 (0.90-5.53)	0.085
**Other cardiovascular disease**	4.24 (2.07-6.42)	0.42 (0.20-0.65)	2.89 (1.61-4.17)	1.49 (0.74-3.01)	0.268
**Central nervous system disease**	0.30 (0-0.89)	0.03 (0-0.09)	0.46 (0-0.97)	0.66 (0.07-6.38)	0.720
**Mental health disorders**	15.36 (11.23-19.49)	1.61 (1.14-2.08)	12.85 (10.11-15.58)	1.23 (0.82-1.84)	0.309
**Eye disorders**	4.60 (2.33-6.88)	0.45 (0.22-0.68)	3.66 (2.23-5.10)	1.27 (0.66-2.45)	0.480
**BMD alterations**	6.38 (3.74-9.02)	0.64 (0.37-0.92)	5.72 (3.93-7.51)	1.12 (0.65-1.95)	0.678
**Fracture**	0.30 (0-0.89)	0.03 (0-0.09)	3.02 (1.71-4.32)	0.10 (0.01-0.72)	0.023
**Cancer**	5.64 (3.11-8.17)	0.57 (0.31-0.83)	7.39 (5.36-9.42)	0.75 (0.43-1.31)	0.313

Data are the value of the parameter indicated with the 95% confidence interval in parentheses.

BMD, bone mineral density.

**Figure 1:**
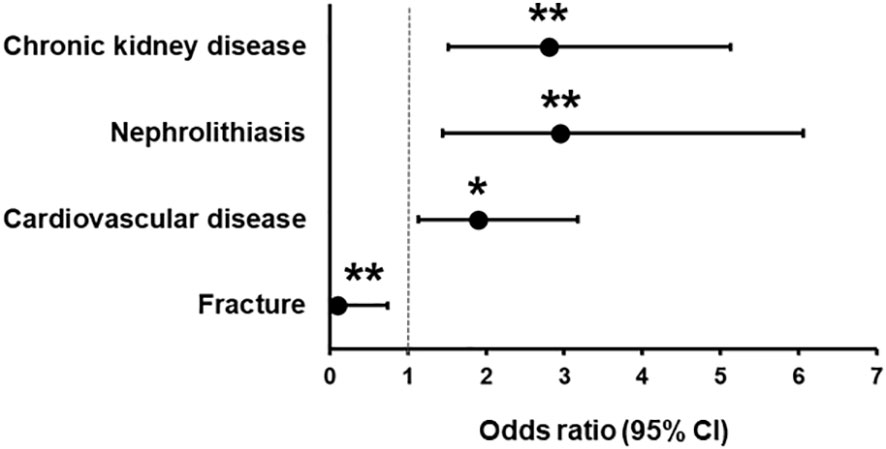
Odds ratio (with 95% confidence intervals) in patients with hypoparathyroidism in comparison with patients without hypoparathyroidism for chronic kidney disease, nephrolithiasis, cardiovascular disease, and fracture. *P < 0.05, **P < 0.01.

When analyzing the association of hypoparathyroidism with the appearance of incident diseases, we found that, in comparison with group NH, patients in group H exhibited a significantly higher OR for the incidence of chronic kidney disease (2.94[1.56-5.55]; P=0.001), nephrolithiasis (3.07[1.46-6.45]; P=0.003) and cardiovascular disease (1.98[1.15-3.42]; P=0.014), and a significantly lower relative risk for fracture (0.10[0.01-0.72]; P=0.023) (**Table 2**; **Figure 1**). When each of the considered cardiovascular diseases were studied individually (i.e., coronary heart disease, cerebrovascular disease, and others), no significant increase in the OR was observed in patients with hypoparathyroidism (**Table 2**).

### Survival analysis

**Figure 2:**
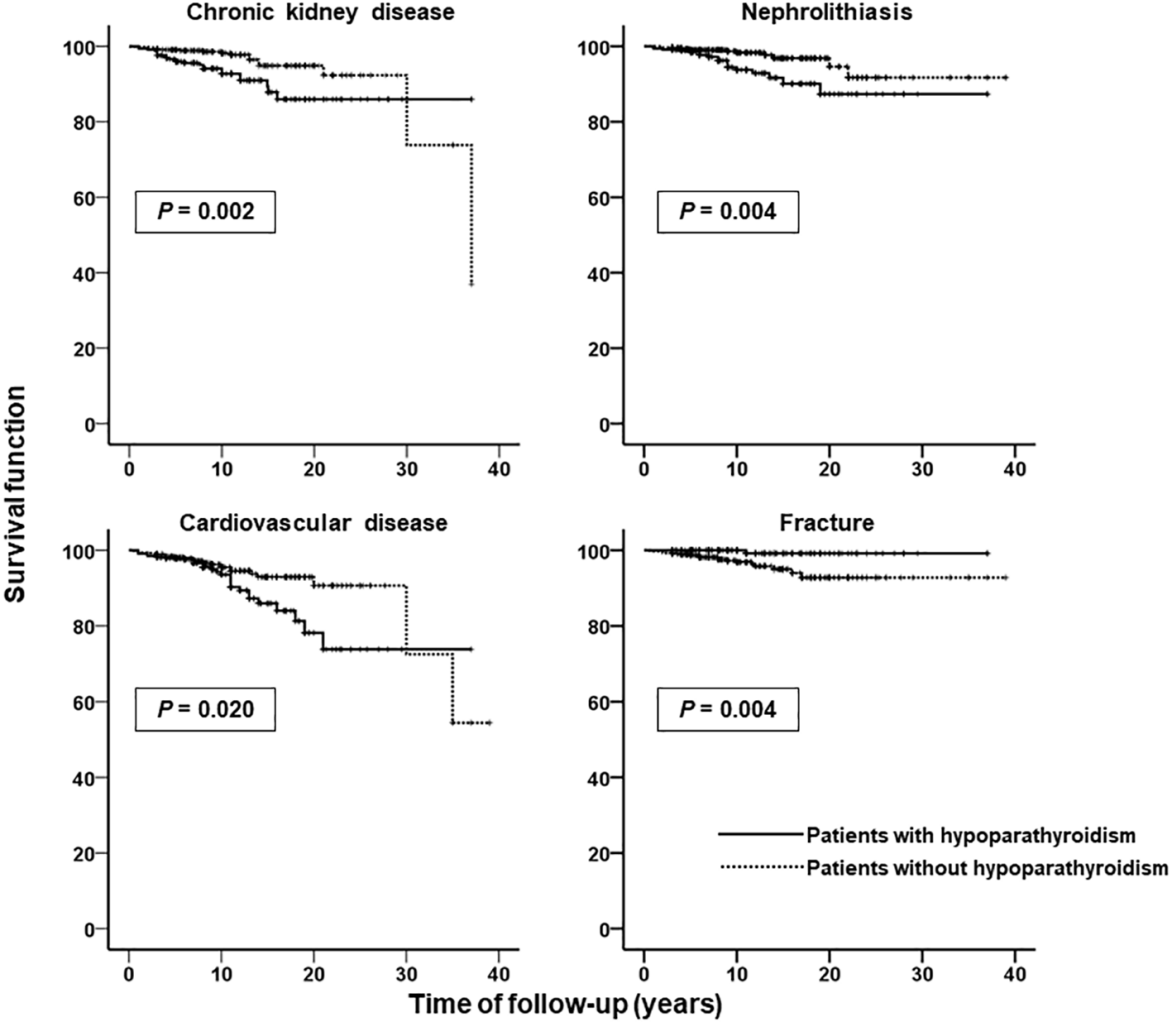
Kaplan-Meier curves for time of follow-up without developing incident chronic kidney disease, nephrolithiasis, cardiovascular disease, and fracture in patients with (solid lines) and without (dashed lines) hypoparathyroidism. Ordinate scale: survival function (proportion of patients not developing incident disease). Abscissa scale: time of follow-up (years).

To assess the influence of hypoparathyroidism for the development of incident comorbidities, we performed a survival analysis using the Kaplan-Meier curves. Only the four incident comorbidities in which a significant increase or reduction in OR was detected in patients with hypoparathyroidism were analyzed (**Figure 2**). Survival free of incident disease was significantly lower in group H regarding chronic kidney disease (P=0.002), nephrolithiasis (P=0.004) and cardiovascular disease (P=0.020). However, incident fracture-free survival was significantly higher in group H compared to group NH (P=0.004).

### Multivariable logistic regression analysis

**Figure 3:**
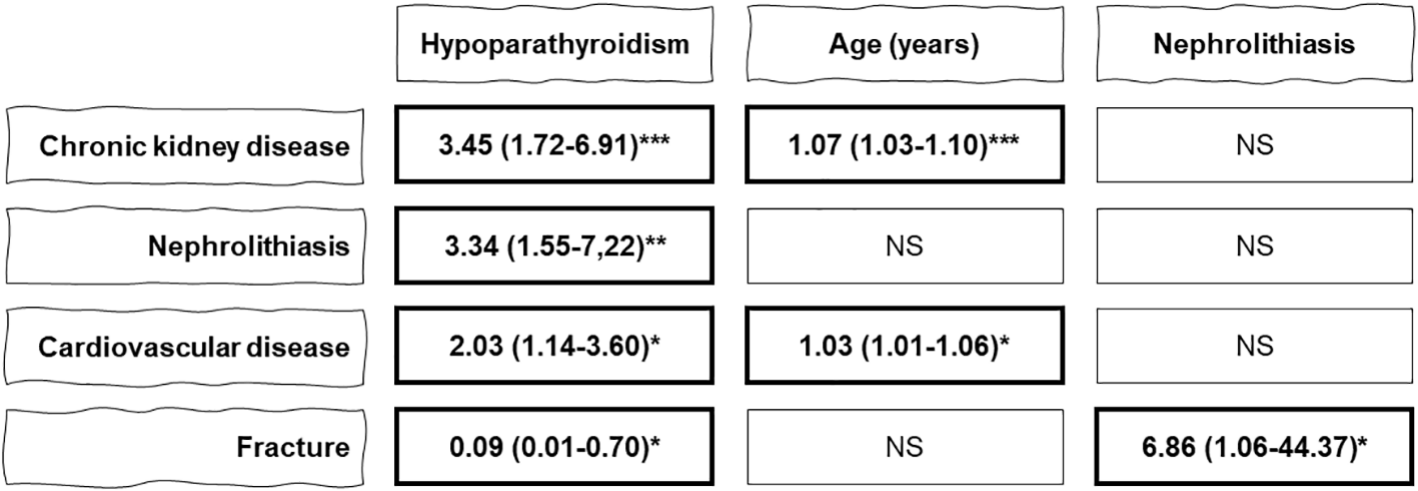
Summary of the results of the multivariable logistic regression analysis (model 2) to study the influence of hypoparathyroidism and different variables on the development of incident comorbidities (chronic kidney disease, nephrolithiasis, cardiovascular disease, and facture). Data are the odds ratio with 95% confidence intervals. NS, non-significant; *P < 0.05, **P < 0.01, ***P < 0.001.

Results of multivariable logistic regression analysis are shown in **Supplementary Tables 4**–**7 (Supplementary Material)**. A summary of the most relevant findings is shown in **Figure 3**. This multivariable analysis, both in model 1 and model 2, showed statistically significant association of chronic kidney disease with the presence of hypoparathyroidism (OR in model 2, 3.45[1.72-6.91]; P<0.001), and advanced age (OR in model 2, 1.07[1.03-1.10]; P<0.001). In the case of nephrolithiasis, the only factor significantly related in the multivariate analysis was hypoparathyroidism (OR in model 2, 3.34[1.55-7.22]; P=0.002). Incident cardiovascular disease was significantly related to hypoparathyroidism (OR in model 2, 2.03[1.14-3.60]; P=0.016) and age (OR in model 2, 1.03[1.01-1.06]; P=0.010). Lastly, our analysis showed that the risk of fracture was significantly increased in patients with nephrolithiasis (OR in model 2, 6.86[1.06-44.37]; P=0.043) but reduced in patients with hypoparathyroidism (OR in model 2, 0.09[0.01-0.70]; P=0.021).

### Pharmacological treatments

No significant differences were found between both groups in the drug categories studied before thyroidectomy. As expected, at the last visit, patients in group H had a significantly greater use of drugs related to the treatment of hypoparathyroidism, i.e., calcium, calcitriol, and thiazide diuretics, as well as a slight but significant lower use of angiotensin-converting enzyme (ACE) inhibitors. The proportions of use of most pharmacological categories increased significantly in both groups, without clinically relevant differences. However, the use of insulin, antiarrhythmic and antipsychotic agents did not increase significantly in any group. Finally, the use of non-thiazide diuretics and calcium antagonists increased significantly only in the NH group (**Table 3**).

**Table 3:** Pharmacological treatments used by patients before thyroidectomy and at the last follow-up visit.

	Before thyroidectomy			Last visit			Comparison last visit vs. before surgery	
	Group H	Group NH		Group H	Group NH		Group H	Group NH
			P			P	P	P
Calcium	9 (2.7)	17 (2.5)	0.526	296 (87.8)	57 (8.5)	<0.001	<0.001	<0.001
Calcitriol				332 (98.5)	0 (0)	<0.001		
Levothyroxine	32 (9.5)	44 (6.6)	0.066	337 (100)	669 (100)			
Vitamin D	21 (6.2)	26 (3.9)	0.068	138 (40.9)	238 (35.6)	0.098	<0.001	<0.001
Hypolipidemic drugs	48 (14.2)	101 (15.1)	0.394	91 (27.0)	200 (29.9)	0.337	<0.001	<0.001
Oral antidiabetics	13 (3.9)	35 (5.2)	0.211	32 (9.5)	67 (10.0)	0.824	<0.001	<0.001
Injectable antidiabetics	2 (0.6)	4 (0.6)	0.677	10 (3.0)	18 (2.7)	0.840	0.008	0.001
Insulin	3 (0.9)	9 (1.3)	0.387	5 (1.5)	15 (2.2)	0.482	0.500	0.109
Thiazide diuretics	16 (4.7)	32 (4.8)	0.562	65 (19.3)	93 (13.9)	0.028	<0.001	<0.001
Non thiazide diuretics	11 (3.3)	12 (1.8)	0.108	19 (5.6)	37 (5.5)	1.000	0.057	<0.001
Beta-blockers	11 (3.3)	27 (4.0)	0.339	29 (8.6)	51 (7.6)	0.622	<0.001	<0.001
Calcium antagonists	20 (5.9)	28 (4.2)	0.142	30 (8.9)	64 (9.6)	0.819	0.064	<0.001
ACE inhibitors	36 (10.7)	91 (13.6)	0.111	70 (20.8)	181 (27.1)	0.031	<0.001	<0.001
Antiarrhythmics	1 (0.3)	4 (0.6)	0.457	4 (1.2)	9 (1.3)	1.000	0.375	0.227
Oral anticoagulants	1 (0.3)	6 (0.9)	0.259	8 (2.4)	14 (2.1)	0.821	0.016	0.039
Anxiolytics	35 (10.4)	63 (9.4)	0.350	62 (18.4)	130 (19.4)	0.734	<0.001	<0.001
Antidepressants	18 (5.3)	43 (6.4)	0.298	38 (11.3)	87 (13.0)	0.479	0.001	<0.001
Antipsychotics	4 (1.2)	9 (1.3)	0.547	5 (1.5)	14 (2.1)	0.627	1.000	0.125

Data are the number of patients (percentage) in each pharmacological group.

ACE, angiotensin-converting enzyme.

## Discussion

The results of the present study show that, in real-life clinical practice in Spain, patients with permanent postsurgical hypoparathyroidism have a higher risk of developing chronic kidney disease, nephrolithiasis, and cardiovascular disease, compared to thyroidectomized patients with normal parathyroid function. On the contrary, hypoparathyroidism seems to reduce the risk of incident fracture. Our data do not show any influence of hypoparathyroidism on other chronic conditions such as hypertension, dyslipidemia, diabetes, mental health disorders, eye disorders, BMD alterations or cancer.

Chronic hypoparathyroidism is associated with different complications that affect multiple organ systems. A recent systematic review (20) recognizes the presence of cataracts in 17% of patients, nephrocalcinosis/nephrolithiasis in 15%, renal failure in 12%, depression in 12%, seizures in 11%, infection in 11%, ischemic heart disease in 7% and arrhythmias in 7%. Most of these prevalence data have been obtained from registry or population-based studies (13–15, 21, 22). Our study has used a different methodology and should be understood as a real-life analysis of the comorbidities presented by patients with long-term chronic hypoparathyroidism and that are diagnosed in the clinical practice of Spanish endocrinologists. To correctly interpret and analyze these results, we must take into account that the clinical guidelines do not clearly establish the necessary examinations for the detection of comorbidities in hypoparathyroidism and that, therefore, most Spanish endocrinologists do not carry out a formal assessment of all possible complications in clinical practice. This fact has recently become evident in an international survey of expert endocrinologists that showed that most of them do not monitor intracerebral calcifications, ophthalmological examination for cataract or BMD on a regular basis (23).

Renal conditions are among the most common complications in patients with hypoparathyroidism (21). The prevalence of chronic kidney disease in patients with hypoparathyroidism ranges from 2.5 to 41% (7, 24) and nephrolithiasis/nephrocalcinosis occurs in 19-31% of patients (13, 24, 25). Our data obtained in clinical practice differ from the findings of previous population-based and registry studies (16, 22, 26, 27). Our results in group H showed that 7.27% of patients developed chronic kidney disease and 5.41% presented with nephrolithiasis throughout the follow-up. In patients with normal parathyroid function these values were only 2.60% for renal failure and 1.83% for nephrolithiasis. The incidence rate was 0.73 cases per 100 patient-years for chronic kidney disease and 0.54 cases per 100 patient-years for nephrolithiasis. Similar to that reported in other studies (13), our multivariate analysis shows that patients with hypoparathyroidism, compared to the NH group, have a three-fold increased risk for chronic kidney disease (OR 3.45; 95% CI, 1.72-6.91) and nephrolithiasis (OR 3.34; 95% CI, 1.55-7.22). Age was also a factor significantly related to chronic kidney disease, while for nephrolithiasis the only factor was hypoparathyroidism.

Hypoparathyroidism may increase the risk of hypercalciuria due to the lack of tubular calcium reabsorption by PTH. Furthermore, treatment with large doses of calcium and active vitamin D can also increase the risk of hypercalciuria, renal stones, and renal insufficiency (25, 28). An increase in the calcium-phosphorus product, together with the deposit of calcium phosphate has been implicated in the increase of renal failure and nephrolithiasis in patients with hypoparathyroidism (13, 27). The number of episodes of hypercalcemia and the duration of disease have also been considered risk factors associated with kidney disease (26).

One of the most noteworthy findings of our study is the increased risk of overall cardiovascular disease in patients with hypoparathyroidism (OR in multivariable analysis, 2.03; 95% CI, 1.14-3.60). Although the subgroup analysis did not show an increase in risk in each of the considered conditions, 8.26% of our patients developed some type of cardiovascular disease during follow-up. Our results contrast with those found in a study of 688 patients with postsurgical hypoparathyroidism identified in a Danish national registry by Underbjerg et al. (13). Compared with controls, these patients did not have an increased risk of cardiovascular disease, cardiac arrhythmias or death. However, in a subsequent study, conducted in 180 patients with nonsurgical hypoparathyroidism, these authors (15) found a significantly increased risk of cardiovascular disease (HR 1.91; 95% CI, 1.29-2.81), similar to that found in the present survey. The population-based study by Vadiveloo et al. (21) showed that nonsurgical hypoparathyroid patients had increased risk of cardiovascular (HR 2.18; 95% CI, 1.41-3.39) and cerebrovascular disease (HR, 2.95, 95% CI, 1.46-5.97). A national population-based Korean study (22) also showed that patients with nonsurgical hypoparathyroidism had a higher risk of cardiovascular disease, especially arrhythmia (HR, 2.03; 95% CI, 1.11-3.70) and heart failure (HR, 2.43; 95% CI, 1.22-4.83). Similarly, a retrospective cohort study using a large medical insurance database in USA showed that patients with chronic hypoparathyroidism had significantly higher risk of incident cardiovascular conditions compared with those without hypoparathyroidism (HR 1.63; 95% CI, 1.52-1.75) (29).

Chronic hypocalcemia and the lack of action of PTH at the cardiac and vascular levels have been implicated as causal factors in cardiovascular complications. In an elegant study, Underbjerg et al. (26) have shown that disturbances in calcium-phosphate homeostasis are significantly associated with risk of complications. In particular, the increased cardiovascular risk was associated with an increased number of hypercalcemic episodes, lower time-weighted serum ionized calcium, and longer duration of hypoparathyroidism.

We analyzed changes in the drug therapies of the two groups of patients. The use of drugs in the baseline situation prior to surgery was very similar between both groups. As expected, at last visit, the use of drugs related to hypoparathyroidism was significantly higher in group H. However, the use of remaining drugs at last visit was similar in both groups, with the exception of a higher proportion of ACE inhibitor users in group NH (group (27.1 vs. 20.8%; P=0.031). The use of calcium and vitamin D has been linked to a possible increase in cardiovascular risk, although study results have been conflicting (13). Our data suggest, but do not prove, that the lower use of ACE inhibitors in group H could be related to the increased risk of cardiovascular disease, although this relationship is uncertain.

On the other hand, our study did not show any relationship between hypoparathyroidism and some of the classic risk factors for cardiovascular disease, such as diabetes, dyslipidemia, and hypertension. We have not found any previous studies showing associations of chronic hypoparathyroidism with hypertension or dyslipidemia. Nevertheless, a recent retrospective database report suggests that chronic hypoparathyroidism is associated with an increased risk of type 2 diabetes (HR 1.80; 95% CI, 1.64-1.96) (30). Further research is needed to confirm these results and understand the potential mechanisms of this association.

It has been well established that PTH deficiency is accompanied by a reduction in bone turnover and abnormalities in skeletal microstructure, both in cortical and cancellous compartments (31–33). An increase in BMD in patients with hypoparathyroidism compared to individuals matched for age and sex has been reported (25, 32, 34). This increase in BMD, in general, affects all skeletal sites, with higher values in the lumbar spine (33). It is not, however, well established whether this increase in BMD is accompanied by a decrease in the risk of fractures, since the available studies have shown contradictory results (14–16, 21, 22, 35, 36). Apart from BMD, other risk factors for fractures in patients with hypoparathyroidism, such as impairment in the trabecular microarchitecture, should be considered. A recent study has shown that bone marrow adipose tissue is increased in postmenopausal women with postsurgical hypoparathyroidism and negatively associated with trabecular microarchitecture (37).

On the other side, it should be emphasized that the evaluation of clinical fractures is not a sensitive method and, therefore, is not the ideal procedure to study the incidence of skeletal health problems in patients with hypoparathyroidism. Recent data showed that a morphometric approach is essential for evaluating bone health in patients with endocrine disorders known to affect skeletal health (38). Therefore, the proactive search of clinically asymptomatic fractures by this method has been recognized as one of the most useful tools in these patients (38). In fact, in a study carried out on 50 postmenopausal women with chronic hypoparathyroidism and 40 age-matched healthy postmenopausal women, Cipriani et al. (36) demonstrated that, although BMD values were higher in the hypoparathyroid group in comparison to healthy controls, patients exhibited a higher incidence of asymptomatic skeletal fractures at vertebral spine.

Furthermore, clinical guidelines do not give precise indications (4) or recommend against routine BMD monitoring (3). However, the detection of incident alterations in BMD in 6.38% of patients in group H and 5.72% of patients in group NH suggests that this examination is frequently used in the clinical practice of Spanish endocrinologists. Although our data did not show statistically different changes in BMD between both groups, a lower risk of incident fracture in patients with hypoparathyroidism compared to subjects with normal calcium metabolism was found. This finding is in line with a recent study that have shown that prevalence of fragility fractures was low in women with hypoparathyroidism and compatible with low fracture risk estimated by the FRAX tool (39). Taken together, these data suggest, although they do not demonstrate, that hypoparathyroidism protects against fracture risk (32). Nonetheless, these data should be taken with caution, because the incidence of fractures was not actively sought by the researchers of this retrospective study and it is possible that some cases of fractures were not detected in clinical practice. Our study also showed a strong association of nephrolithiasis with the incidence of fractures. This data is difficult to explain and could be related to the small number of events recorded in group H.

Mental health disorders, including depression, anxiety and bipolar affective disorder have been reported in patients with hypoparathyroidism (14, 21, 22). Our survey detected that 15% of patients with hypoparathyroidism had mental health disorders throughout the follow-up. This cumulative incidence was slightly higher than that found in group NH, although it did not reach statistical significance. The detection of psychiatric problems is common in patients with hypoparathyroidism and has been related to a decrease in the quality of life of these patients. Different studies have been able to demonstrate a significant negative impact on mental and emotional health using instruments validated for chronic diseases (40–42) and also disease-specific instrument developed and validated for hypoparathyroidism (43). As with tumor development, a protective role of vitamin D for neurocognitive disorders is possible. In fact, some data suggest that vitamin D is important for normal brain development and function in rodents and humans (44). In a recent study, carried out in coronoavirus disease-19 (COVID) survivors with long COVID, lower 25(OH)-vitamin D levels were observed in those with neurocognitive symptoms at follow-up than those without (45). However, vitamin D supplements have produced conflicting results on neurocognitive performance (44).

Although our patients with hypoparathyroidism had a higher proportion of eye disorders during follow-up (4.60%), the difference with patients in the NH group (3.66%) was not significant. The increased risk of cataract in hypoparathyroidism has been well documented (15, 20–22) and has been related to the duration of disease (16, 22). Similarly, we have also not found an increased risk of central nervous system diseases even though the prevalence of basal ganglia calcifications has been reported in 37% of nonsurgical patients and in 15% of postsurgical patients (46), and the risk of epilepsy has been found to be elevated in nonsurgical and surgical hypoparathyroidism (13, 20–22).

The detection of complications is clearly dependent on carrying out an active search. In the study by Mitchell et al. (25), of those patients with renal imaging, 31% had renal calcifications, and 52% of those with head imaging had basal ganglia calcifications. The lack of detection of an increased risk of cataract or central nervous system conditions can be explained because this is a retrospective study of routine clinical practice and, in our country, there are no protocols or clinical guidelines on screening for these chronic complications of hypoparathyroidism. Unfortunately, our data suggest that most of Spanish endocrinologists do not actively search for cases of cataract or central nervous system disease (47). Nevertheless, it is worth mentioning that basal ganglia calcification was not identified as one of the common complications of hypoparathyroidism in a recent systematic review of observational studies (7).

Our data do not provide evidence of a higher incidence of malignancies in patients with hypoparathyroidism. We must assume that in our study population no specific detection tests are performed on these patients, but only general population cancer screening. Our results agree with those from the Danish registry study, which showed that the risk of overall malignant diseases did not differ between patients with postsurgical hypoparathyroidism and controls (14). Nonetheless, the risk of gastrointestinal cancers was significantly lower in patients in this study (14) and the risk of overall malignancy was decreased among patients with nonsurgical hypoparathyroidism (15). This cancer risk reduction has been attributed to the use of calcium and vitamin D in these patients, since there is an inverse association between vitamin D status, calcium intake and the risk of digestive cancer (14)

Our results may have implications for clinical practice. Prevalence of complications of chronic hypoparathyroidism may vary among patient populations and the methodology used. Our findings, based on clinical practice, could provide useful information for future guidelines and consensus on the practical management of patients with hypoparathyroidism. The increased risk of cardiovascular disease registered in our analysis might explain the increased mortality in hypoparathyroidism reported in some epidemiological studies (21, 48). However, this aspect is not conclusive, since other studies have not detected an increase in mortality (13, 15, 22).

Monitoring of complications of chronic hypoparathyroidism is not well established and the recommendations offered by the guidelines are based on expert opinions and consensus statements (23). Our study highlights the long-term morbidity associated with hypoparathyroidism found in real clinical practice by Spanish endocrinologists. We suggest that a more active and rigorous monitoring of hypoparathyroidism comorbidities will lead to greater detection of complications and will have an impact on the epidemiology of the disease and the prognosis of patients.

The main strengths of our study include the high sample size, taking into account the rarity of the disease, and its multicenter and nationwide design, as well as the non-inclusion of patients with hypoparathyroidism of short duration (<3 years). Our investigation includes diagnoses made in real clinical practice by expert specialists. Although all diagnoses are reliable and are recorded in the patients’ medical records, it is possible that there are unrecorded diagnoses and, therefore, comorbidities not detected in this study. Additionally, in our study, the two groups studied were comparable at baseline not only in age, sex and time of evolution, but also in prevalent disease burden and use of drugs.

Among the limitations, we must point out that our study included a cohort with selection of a non-exposed group that is not representative of the total. However, our non-exposed group (group NH) can be considered at higher risk of developing comorbidities, since they are patients with hospital follow-up. Our study required the included patients to be alive at the time of the study, that is, it presents an immortal time bias. However, our study did not aim to analyze mortality and, furthermore, patients have a mean age (45 years) at which deaths are not expected in the short term. Although our sample size is noteworthy, it may not have a sufficient size to detect comorbidities with a low incidence. In the particular case of fractures, we have to recognize that the use of anamnesis or records of fractures with clinical manifestation are not the most appropriate procedures to investigate the impact of hypoparathyroidism on the skeleton. We do not have data on quality of life or the incidence of infections, aspects of clinical interest in these patients. Another limitation is that our study design did not include smoking and, therefore, we cannot analyze the effect of smoking on incident comorbidities in patients with hypoparathyroidism. Further to this, our study was limited to the setting of specialized medical care in Spain, so the results could vary in different settings or countries.

In summary, to our knowledge, this is the first study that analyzes a large number of incident comorbidities in patients with chronic hypoparathyroidism using clinical practice data. The results are consistent with the associations found in large-scale database and registry analysis. However, some results from clinical practice do not agree with registry studies. This may be due to the lack of agreed criteria for exhaustive screening of complications in chronic hypoparathyroidism. We believe that further real-life studies are necessary to inform the writing of future clinical guidelines and monitoring protocols.

## Data availability statement

The original contributions presented in the study are included in the article/**Supplementary Material**. Further inquiries can be directed to the corresponding author.

## Ethics statement

The patient’s confidential information was protected according to national law, and the study received favorable report from the ethics committee of the Hospital Universitario Puerta de Hierro Majadahonda (PI 253/22). Full name and affiliation: Belén Ruiz Antorán, Hospital Universitario Puerta de Hierro Majadahonda, Calle Joaquín Rodrigo 2, 28222 Majadahonda (Madrid, Spain). Phone: +34911916000. The studies were conducted in accordance with the local legislation and institutional requirements. The ethics committee/institutional review board waived the requirement of written informed consent for participation from the participants or the participants’ legal guardians/next of kin because all patient’s data were obtained under the standard medical care conditions. This is a retrospective study without participation of any patients.

## Author contributions

JD: Conceptualization, Data curation, Formal analysis, Investigation, Methodology, Project administration, Software, Supervision, Validation, Visualization, Writing – original draft, Writing – review & editing. EA: Data curation, Investigation, Methodology, Supervision, Validation, Visualization, Writing – review & editing. BP-C: Data curation, Investigation, Methodology, Supervision, Validation, Visualization, Writing – review & editing. MPa: Data curation, Investigation, Methodology, Supervision, Validation, Visualization, Writing – review & editing. VA: Data curation, Investigation, Methodology, Supervision, Validation, Visualization, Writing – review & editing. CS-R: Data curation, Investigation, Methodology, Supervision, Validation, Visualization, Writing – review & editing. AOr: Data curation, Investigation, Methodology, Supervision, Validation, Visualization, Writing – review & editing. AR-L: Data curation, Investigation, Methodology, Supervision, Validation, Visualization, Writing – review & editing. MS: Data curation, Investigation, Methodology, Supervision, Validation, Visualization, Writing – review & editing. AOl: Data curation, Investigation, Methodology, Supervision, Validation, Visualization, Writing – review & editing. ÁC: Data curation, Investigation, Methodology, Supervision, Validation, Visualization, Writing – review & editing. MA: Data curation, Investigation, Methodology, Supervision, Validation, Visualization, Writing – review & editing. VU: Data curation, Investigation, Methodology, Supervision, Validation, Visualization, Writing – review & editing. AD-L: Data curation, Investigation, Methodology, Supervision, Validation, Visualization, Writing – review & editing. JF-G: Data curation, Investigation, Methodology, Supervision, Validation, Visualization, Writing – review & editing. VK-D: Data curation, Investigation, Methodology, Supervision, Validation, Visualization, Writing – review & editing. SD-D: Writing – review & editing. TM: Data curation, Investigation, Methodology, Supervision, Validation, Visualization, Writing – review & editing. MPe: Data curation, Investigation, Methodology, Supervision, Validation, Visualization, Writing – review & editing. JS: Conceptualization, Data curation, Formal analysis, Investigation, Methodology, Supervision, Validation, Visualization, Writing – review & editing.
