# Supplementary material for: Incident comorbidities in patients with chronic hypoparathyroidism after thyroidectomy: a multicenter nationwide study
Source: Front Endocrinol (Lausanne). 2024 Feb 28;15:1348971. doi: 10.3389/fendo.2024.1348971 (PMC10936239; doi:10.3389/fendo.2024.1348971)
Supplement: Supplementary file 1 [file Table_1.docx]

**Incident comorbidities in patients with chronic hypoparathyroidism after thyroidectomy: a multicenter nationwide study**

**SUPPLEMENTARY MATERIAL**

**Table S1.** Characteristics of patients with hypoparathyroidism: replacement therapies used at the time of the last visit

|  | **Patients on therapy** | | **Drug dose** | | |
| --- | --- | --- | --- | --- | --- |
|  | **Number** | **Percentage** | **Median** | **IQR** | **Units** |
| **Calcium** | 296 | 87.8 | 1250 | 1000-2000 | mg/day |
| **Calcitriol** | 332 | 98.5 | 0.50 | 0.25-0.50 | μg/day |
| **Levothyroxine** | 337 | 100 | 125 | 100-150 | μg/day |
| **Vitamin D** | 138 | 40.9 |  |  |  |

Abbreviations: IQR, interquartile range.

**Table S2.** Characteristics of patients with hypoparathyroidism: laboratory values related to disease control at the time of the last visit

|  | **N** | **Median** | **IQR** | **Number** | **percentage** |
| --- | --- | --- | --- | --- | --- |
| **Calcium, mg/dl** | 337 | 8.7 | 8.3-9.1 |  |  |
| **Phosphorus, mg/dl** | 325 | 4.3 | 3.8-4.8 |  |  |
| **Ca x P, mg^2^/dl^2^** | 235 | 36.6 | 32.4-40.3 |  |  |
| **Calciuria, mg/24 h** | 152 | 204.8 | 134.3-268.4 |  |  |
| **25(OH)D, ng/ml** | 250 | 29.7 | 22.0-38.0 |  |  |
| **Thyrotropin** | 335 | 1.10 | 0.36-2.72 |  |  |
| **Creatinine, mg/dl** | 335 | 0.80 | 0.72-0.94 |  |  |
| **eGFR, ml/min/1.73m^2^** | 306 | 84.0 | 71.8-90.0 |  |  |
| **Calcium ≥8.0 mg/dl** | 337 |  |  | 297 | 88.1 |
| **Phosphorus ≤4.5 mg/dl** | 325 |  |  | 213 | 65.5 |
| **Ca x P <55 mg^2^/dl^2^** | 235 |  |  | 320 | 98.5 |
| **Calciuria <250 mg/24 h (female) or <300 mg/24h (male)** | 152 |  |  | 112 | 73.7 |

Abbreviations: N, number of patients in which the parameter is available; IQR, interquartile range; Ca x P, serum calcium–phosphate product; 25(OH)D, 25-hydroxyvitamin D; eGFR, estimated glomerular filtration rate.

**Table S3**. Row data in patients with and without hypoparathyroidism regarding incident comorbidity

|  | **Patients with hypoparathyroidism** | | | **Patients without hypoparathyrodism** | |
| --- | --- | --- | --- | --- | --- |
|  | **New cases** | **Patients at risk** | **Patient-years** | **New cases** | **Patients at risk** |
| **Chronic kidney disease** | 24 | 330 | 3296.1 | 17 | 654 |
| **Nephrolithiasis** | 18 | 333 | 3348.9 | 12 | 656 |
| **Hypertension** | 43 | 282 | 2722.2 | 107 | 548 |
| **Dyslipidemia** | 55 | 272 | 2573.3 | 117 | 530 |
| **Diabetes** | 23 | 322 | 3178.3 | 42 | 628 |
| **Cardiovascular disease** | 27 | 327 | 3209.4 | 28 | 644 |
| **Coronary heart disease** | 7 | 334 | 3389.7 | 8 | 661 |
| **Other CVD** | 14 | 330 | 3303.0 | 19 | 657 |
| **Cerebrovascular disease** | 10 | 336 | 3370.2 | 9 | 662 |
| **CNS disease** | 1 | 331 | 3386.9 | 3 | 657 |
| **Mental health disorders** | 45 | 293 | 2801.1 | 74 | 576 |
| **Eye disorders** | 15 | 326 | 3299.3 | 24 | 655 |
| **BMD alterations** | 21 | 329 | 3270.1 | 37 | 647 |
| **Fracture** | 1 | 334 | 3395.0 | 20 | 663 |
| **Cancer** | 18 | 319 | 3173.8 | 47 | 636 |

Abbreviations: CVD, cardiovascular disease; CNS, central nervous system; BMD, bone mineral density.

**Table S4**. Results of two models* of multivariable logistic regression analysis to study the influence of several covariates on the development of incident chronic kidney disease

|  | **Model 1** | | **Model 2** | |
| --- | --- | --- | --- | --- |
|  | **OR (95% CI)** | **P** | **OR (95% CI)** | **P** |
| **Hypoparathyroidism** | **3.38 (1.70-6.71)** | **0.001** | **3.45 (1.72-6.91)** | **<0.001** |
| **Gender, male** | 0.65 (0.23-1.81) | 0.409 | 0.71 (0.25-1.99) | 0.511 |
| **Age, yr** | **1.07 (1.04-1.10)** | **<0.001** | **1.07 (1.03-1.10)** | **<0.001** |
| **Thyroidectomy, 2-stage** | 1.88 (1.75-4.68) | 0.178 | 1.95 (0.77-4.98) | 0.162 |
| **Thyroid cancer** | 0.76 (0.35-1.57) | 0.454 | 0.70 (0.33-1.49) | 0.356 |
| **Hypertension** | 1.89 (0.83-4.34) | 0.132 | 1.90 (0.81-4.43) | 0.138 |
| **Dyslipidemia** | 0.72 (0.31-1.67) | 0.442 | 0.66 (0.28-1.55) | 0.337 |
| **Diabetes** | 2.42 (0.76-7.68) | 0.134 | 2.60 (0.80-8.50) | 0.114 |
| **Cardiovascular disease** | 2.07 (0.55-7.76) | 0.283 | 2.06 (0.55-7.72) | 0.285 |
| **BMD alterations** | 0.40 (0.05-3.23) | 0.391 | 0.30 (0.03-2.88) | 0.297 |
| **Nephrolithiasis** |  |  | 1.72 (0.26-11.57) | 0.578 |
| **CNS disease** |  |  | 3.11 (0.57-16.98) | 0.190 |
| **Mental health disorders** |  |  | 1.11 (0.46-2.70) | 0.813 |
| **Eye disorder** |  |  | 1.48 (0.33-6.58) | 0.609 |
| **Fracture** |  |  | 5.03 (0.48-52.90) | 0.179 |
| **Cancer** |  |  | 1.07 (0.27-4.23) | 0.920 |

Abbreviations: OR, odds ratio; CI, confidence interval; BMD, bone mineral density; CNS, central nervous system.

*Two models of multivariate analysis have been used: model 1, adjusted for the presence of hypoparathyroidism, gender, age, thyroidectomy, histopathology, hypertension, dyslipidemia, diabetes, cardiovascular disease, and BMD alterations; model 2, adjusted for the same covariates, and nephrolithiasis, CNS disease, mental health disorders, eye disorders, fracture and cancer.

**Table S5**. Results of two models* of multivariable logistic regression analysis to study the influence of several covariates on the development of incident nephrolithiasis

|  | **Model 1** | | **Model 2** | |
| --- | --- | --- | --- | --- |
|  | **OR (95% CI)** | **P** | **OR (95% CI)** | **P** |
| **Hypoparathyroidism** | **3.24 (1.51-6.95)** | **0.003** | **3.34 (1.55-7.22)** | **0.002** |
| **Gender, male** | 1.12 (0.41-3.06) | 0.831 | 1.09 (0.39-3.03) | 0.874 |
| **Age, yr** | 1.01 (0.98-1.05) | 0.416 | 1.02 (0.98-1.05) | 0.386 |
| **Thyroidectomy, 2-stage** | 1.78 (0.69-4.61) | 0.232 | 1.76 (0.68-4.61) | 0.246 |
| **Thyroid cancer** | 1.00 (0.42-2.39) | 0.995 | 1.06 (0.44-2.55) | 0.899 |
| **Hypertension** | 0.56 (0.15-2.06) | 0.383 | 0.56 (0.15-2.05) | 0.382 |
| **Dyslipidemia** | 0.67 (0.21-2.13) | 0.492 | 0.69 (0.22-2.22) | 0.537 |
| **Diabetes** | 1.92 (0.37-9.99) | 0.441 | 2.42 (0.46-12.73) | 0.297 |
| **Cardiovascular disease** | 2.79 (0.54-14.44) | 0.222 | 3.40 (0.64-18.08) | 0.151 |
| **BMD alterations** | 0 (0) | 0.998 | 0 (0) | 0.998 |
| **Chronic kidney disease** |  |  | 0 (0) | 0.998 |
| **CNS disease** |  |  | 0 (0) | 0.998 |
| **Mental health disorders** |  |  | 1.25 (0.41-3.81) | 0.691 |
| **Eye disorder** |  |  | 1.72 (0.21-13.99) | 0.614 |
| **Fracture** |  |  | 0 (0) | 0.999 |
| **Cancer** |  |  | 0 (0) | 0.997 |

Abbreviations: OR, odds ratio; CI, confidence interval; BMD, bone mineral density; CNS, central nervous system.

*Two models of multivariate analysis have been used: model 1, adjusted for the presence of hypoparathyroidism, gender, age, thyroidectomy, histopathology, hypertension, dyslipidemia, diabetes, cardiovascular disease, and BMD alterations; model 2, adjusted for the same covariates, and nephrolithiasis, CNS disease, mental health disorders, eye disorders, fracture and cancer.

**Table S6**. Results of two models* of multivariable logistic regression analysis to study the influence of several covariates on the development of incident cardiovascular disease

|  | **Model 1** | | **Model 2** | |
| --- | --- | --- | --- | --- |
|  | **OR (95% CI)** | **P** | **OR (95% CI)** | **P** |
| **Hypoparathyroidism** | **2.05 (1.17-3.62)** | **0.013** | **2.03 (1.14-3.60)** | **0.016** |
| **Gender, male** | 1.09 (0.50-2.38) | 0.830 | 1.14 (0.51-2.52) | 0.753 |
| **Age, yr** | **1.04 (1.01-1.06)** | **0.006** | **1.03 (1.01-1.06)** | **0.010** |
| **Thyroidectomy, 2-stage** | 1.42 (0.63-3.18) | 0.399 | 1.51 (0.66-3.42) | 0.326 |
| **Thyroid cancer** | 0.67 (0.36-1.25) | 0.207 | 0.61 (0.32-1.15) | 0.126 |
| **Hypertension** | 0.93 (0.43-2.03) | 0.860 | 0.96 (0.44-2.11) | 0.920 |
| **Dyslipidemia** | 1.23 (0.61-2.48) | 0.563 | 1.10 (0.53-2.27) | 0.799 |
| **Diabetes** | 2.46 (0.94-6.45) | 0.066 | 2.63 (0.99-6.99) | 0.052 |
| **BMD alterations** | 0.82 (0.18-3.76) | 0.798 | 0.64 (0.13-3.25) | 0.594 |
| **Chronic kidney disease** |  |  | 2.49 (0.59-10.51) | 0.216 |
| **Nephrolithiasis** |  |  | 1.33 (0.16-11.16) | 0.794 |
| **CNS disease** |  |  | 1.84 (0.32-10.60) | 0.495 |
| **Mental health disorders** |  |  | 1.23 (0.58-2.63) | 0.586 |
| **Eye disorder** |  |  | 0 (0) | 0.998 |
| **Fracture** |  |  | 0 (0) | 0.999 |
| **Cancer** |  |  | 2.41 (0.92-6.33) | 0.075 |

Abbreviations: OR, odds ratio; CI, confidence interval; BMD, bone mineral density; CNS, central nervous system.

*Two models of multivariate analysis have been used: model 1, adjusted for the presence of hypoparathyroidism, gender, age, thyroidectomy, histopathology, hypertension, dyslipidemia, diabetes, cardiovascular disease, and BMD alterations; model 2, adjusted for the same covariates, and nephrolithiasis, CNS disease, mental health disorders, eye disorders, fracture and cancer.

**Table S7**. Results of two models of multivariable logistic regression analysis to study the influence of several covariates on the development of incident fracture

|  | **Model 1** | | **Model 2** | |
| --- | --- | --- | --- | --- |
|  | **OR (95% CI)** | **P** | **OR (95% CI)** | **P** |
| **Hypoparathyroidism** | **0.09 (0.01-0.70)** | **0.021** | **0.09 (0.01-0.70)** | **0.021** |
| **Gender, male** | 0.62 (0.14-2.80) | 0.535 | 0.60 (0.13-2.76) | 0.515 |
| **Age, yr** | 1.03 (0.99-1.07) | 0.110 | 1.04 (0.99-1.08) | 0.069 |
| **Thyroidectomy, 2-stage** | 0.98 (0.27-3.57) | 0.970 | 1.01 (0.28-3.72) | 0.985 |
| **Thyroid cancer** | 0.62 (0.23-1.69) | 0.348 | 0.54 (0.19-1.53) | 0.247 |
| **Hypertension** | 0.73 (0.21-2.57) | 0.627 | 0.55 (0.14-2.18) | 0.397 |
| **Dyslipidemia** | 0.89 (0.28-2.88) | 0.850 | 0.67 (0.19-2.36) | 0.537 |
| **Diabetes** | 1.28 (0.22-7.33) | 0.781 | 1.50 (0.25-8.87) | 0.653 |
| **Cardiovascular disease** | 0.93 (0.10-8.38) | 0.950 | 0.60 (0.06-6.32) | 0.666 |
| **BMD alterations** | 1.18 (0.97-18.01) | 0.055 | 2.61 (0.50-13.60) | 0.255 |
| **Chronic kidney disease** |  |  | 2.91 (0.43-19.88) | 0.276 |
| **Nephrolithiasis** |  |  | **6.86 (1.06-44.37)** | **0.043** |
| **CNS disease** |  |  | 0 (0) | 0.998 |
| **Mental health disorders** |  |  | 1.53 (0.50-4.63) | 0.456 |
| **Eye disorder** |  |  | 0 (0) | 0.998 |
| **Cancer** |  |  | 1.63 (0.32-8.35) | 0.559 |

Abbreviations: OR, odds ratio; CI, confidence interval; BMD, bone mineral density; CNS, central nervous system.

*Two models of multivariate analysis have been used: model 1, adjusted for the presence of hypoparathyroidism, gender, age, thyroidectomy, histopathology, hypertension, dyslipidemia, diabetes, cardiovascular disease, and BMD alterations; model 2, adjusted for the same covariates, and nephrolithiasis, CNS disease, mental health disorders, eye disorders, fracture and cancer.
